# 5-HT_7_ receptors as modulators of neuronal excitability, synaptic transmission and plasticity: physiological role and possible implications in autism spectrum disorders

**DOI:** 10.3389/fncel.2014.00250

**Published:** 2014-08-27

**Authors:** Lucia Ciranna, Maria Vincenza Catania

**Affiliations:** ^1^Department of Biomedical Sciences, University of CataniaCatania, Italy; ^2^Institute of Neurological Sciences, the National Research Council of Italy (CNR)Catania, Italy; ^3^Laboratory of Neurobiology, IRCCS Oasi Maria SSTroina, Italy

**Keywords:** serotonin, 5-HT_7_ receptor, synaptic function, autism spectrum disorders, Fragile X Syndrome

## Abstract

Serotonin type 7 receptors (5-HT_7_) are expressed in several brain areas, regulate brain development, synaptic transmission and plasticity, and therefore are involved in various brain functions such as learning and memory. A number of studies suggest that 5-HT_7_ receptors could be potential pharmacotherapeutic target for cognitive disorders. Several abnormalities of serotonergic system have been described in patients with autism spectrum disorder (ASD), including abnormal activity of 5-HT transporter, altered blood and brain 5-HT levels, reduced 5-HT synthesis and altered expression of 5-HT receptors in the brain. A specific role for 5-HT_7_ receptors in ASD has not yet been demonstrated but some evidence implicates their possible involvement. We have recently shown that 5-HT_7_ receptor activation rescues hippocampal synaptic plasticity in a mouse model of Fragile X Syndrome, a monogenic cause of autism. Several other studies have shown that 5-HT_7_ receptors modulate behavioral flexibility, exploratory behavior, mood disorders and epilepsy, which include core and co-morbid symptoms of ASD. These findings further suggest an involvement of 5-HT_7_ receptors in ASD. Here, we review the physiological roles of 5-HT_7_ receptors and their implications in Fragile X Syndrome and other ASD.

## Introduction

The monoamine serotonin (5-HT) is widely distributed in the central nervous system (CNS), where it functions as neurotransmitter and neuro-hormone to control mood, circadian rhythm, nociception, hormone secretion, feeding and sexual behavior (Hannon and Hoyer, 2008; Nichols and Nichols, 2008). 5-HT and its receptors are also present in peripheral tissues where they influence several functions including intestinal motility (Foxx-Orenstein et al., 1996), immune/inflammatory response (Ahern, 2011), modulation of nociception (Cervantes-Duran et al., 2013). Seven families of 5-HT receptors have been identified to date in mammals, 5-HT_1_ through 5-HT_7_, each including distinct receptor subtypes. 5-HT_3_ receptors are ligand-gated ion channels mediating fast depolarization (Sugita et al., 1992). All the other 5-HT receptors are G protein-coupled metabotropic receptors: 5-HT_1_ and 5-HT_5_ receptors inhibit adenylate cyclase, 5-HT_4_, 5-HT_6_ and 5-HT_7_ receptors instead stimulate adenylate cyclase, whereas the 5-HT_2_ receptor family is positively linked to phospholipase C (Hannon and Hoyer, 2008; Millan et al., 2008; Pytliak et al., 2011).

5-HT_7_ receptors were the last to be cloned in 1993 by different independent laboratories (Lovenberg et al., 1993; Monsma et al., 1993; Ruat et al., 1993) and their role was initially poorly understood. In the last decade, evidence has emerged that 5-HT_7_ receptors play an important role in the control of body temperature, sleep/wake cycle, nociception, learning and memory (Table 1). 5-HT_7_ receptors are also involved in mood disorders, epilepsy and pain and are starting to be considered as possible targets for these disorders.

**Table 1:** ***In vivo* effects of pharmacological activation, pharmacological blockade or genetic ablation of 5-HT_7_ receptors**.

	***In vivo* effects of 5-HT_7_ receptor manipulation**	**References**
Learning	Genetic ablation of 5-HT_7_ receptors: impairment of fear-related learning and object location memory	Roberts et al. (2004a) and Sarkisyan and Hedlund (2009)
	Pharmacological activation of 5-HT_7_ receptors:	
	– improvement of contextual learning	Eriksson et al. (2008, 2012)
	– improvement of instrumental learning	Perez-Garcia and Meneses (2005)
	– improvement of novelty detection	Freret et al. (2014)
	Pharmacological blockade of 5-HT_7_ receptors: impairment of novelty detection	Ballaz et al. (2007a)
	Pharmacological blockade of 5-HT_7_ receptors: improvement of long-term memory	Gasbarri et al. (2008)
Behavioral flexibility	Pharmacological blockade of 5-HT_7_ receptors: improved behavioral flexibility	Nikiforuk (2012) and Nikiforuk and Popik (2013)
Exploratory activity	Pharmacological activation of 5-HT_7_ receptors:	
	– reduced ambulatory activity	Clissold et al. (2013)
	– enhanced ambulatory activity	Adriani et al. (2012) and Ruocco et al. (2013)
Sleep/wake cycle	Genetic ablation of 5-HT_7_ receptors: reduced REM sleep	Hedlund et al. (2005)
	Pharmacological blockade of 5-HT_7_ receptors: reduced REM sleep	Hagan et al. (2000), Thomas et al. (2003), Bonaventure et al. (2007) and Monti et al. (2012)
	Pharmacological activation of 5-HT_7_ receptors:	
	– reduced REM sleep	Monti et al. (2008, 2014)
	– phase-advance shift of circadian rhythm	Adriani et al. (2012)
Thermoregulation	Pharmacological activation of 5-HT_7_ receptors: hypothermia	Hagan et al. (2000), Guscott et al. (2003) and Hedlund et al. (2003, 2010)
Nociception	Pharmacological activation of 5-HT_7_ receptors: central antinociceptive and peripheral pro-nociceptive effects	Yanarates et al. (2010) and Viguier et al. (2012, 2013)
Mood	Genetic ablation of 5-HT_7_ receptors: antidepressant effects	Hedlund et al. (2005) and Guscott et al. (2005)
	Pharmacological blockade of 5-HT_7_ receptors: antidepressant effects	Hedlund et al. (2005), Wesolowska et al. (2006a) and Mnie-Filali et al. (2011)
	Non-selective 5-HT_7_ receptor antagonists (atypical antipsychotics) behave as antidepressant in humans	Abbas et al. (2009), Guilloux et al. (2013) and Woo et al. (2013)
	Genetic ablation of 5-HT_7_ receptors: no alteration of anxiety	Roberts et al. (2004a) and Guscott et al. (2005)
	Pharmacological blockade of 5-HT_7_ receptors: anxiolytic effects	Wesolowska et al. (2006a,b)
Epilepsy	Pharmacological blockade of 5-HT_7_ receptors: antiepileptic effects against:
	– audiogenic seizures	Bourson et al. (1997)
	– absence epilepsy	Graf et al. (2004)
	– pilocarpine-induced seizures	Yang et al. (2012)
	Pharmacological activation of 5-HT_7_ receptors: antiepileptic effect against picrotoxin-induced seizures	Pericic and Svob Strac (2007)
	Genetic ablation of 5-HT_7_ receptors: reduced sensitivity to electrically- and chemically-induced seizures	Witkin et al. (2007)

The structural, pharmacological and functional features of 5-HT_7_ receptors have been extensively illustrated in recent reviews (Matthys et al., 2011; Gellynck et al., 2013); thus, we will only briefly outline these receptor properties in the first part of the present work. A core part of our review is dedicated to the physiological functions regulated by 5-HT_7_ receptors and particularly to 5-HT_7_ receptor-mediated effects on cognition and mood regulation, two higher brain functions that are strictly related and mutually influence each other. Higher brain functions depend on the activity of brain neuronal networks, which are profoundly affected by changes in neuronal excitability and synaptic efficacy. For this reason, we will describe the effects of 5-HT_7_ receptor activation on intrinsic neuronal excitability, synaptic transmission and synaptic plasticity, and will discuss the possible functional consequences of these 5-HT_7_ receptor-mediated effects.

In the last part, we will review the current literature showing an impairment of the brain serotonin system in autism spectrum disorders (ASD) and its involvement in their pathophysiology. A possible malfunction of 5-HT_7_ receptors in ASD has not yet been investigated. In this respect, we will highlight a number of reports indicating that 5-HT_7_ receptors regulate many physiological functions that are altered in ASD. In addition, we will provide indication from different research groups and from our laboratories that pharmacological manipulation of 5-HT_7_ receptors might be considered as a therapeutic strategy in ASD.

## Characterization of 5-HT_7_ receptors

### Localization, structure and pharmacological profile

5-HT_7_ receptors are highly expressed in the thalamus, hypothalamus and hippocampus of mice and rats; significant amounts were detected in cerebral cortex, amygdala, striatum, cerebellum and spinal cord (Belenky and Pickard, 2001; Neumaier et al., 2001; Bickmeyer et al., 2002; Geurts et al., 2002; Muneoka and Takigawa, 2003; Doly et al., 2005; reviewed by Hedlund and Sutcliffe, 2004).

In the human brain, 5-HT_7_ receptor mRNA was detected in many CNS areas, with highest expression levels in thalamus, hypothalamus, amygdala and hippocampus (Hagan et al., 2000). Autoradiographic studies confirmed that 5-HT_7_ receptor levels in human brain are in good correlation with those found in rodents, with high density in thalamus, dorsal raphe, hippocampus (Martin-Cora and Pazos, 2004; Varnäs et al., 2004) and hypothalamus (Varnäs et al., 2004). Unlike in rodents, in the human brain 5-HT_7_ receptor binding sites were also found at high levels in caudate nucleus, putamen and substantia nigra (Martin-Cora and Pazos, 2004).

In the rodent brain, the immunoreactivity for 5-HT_7_ receptors was high in several regions at birth and then decreased progressively during postnatal development (Muneoka and Takigawa, 2003; García-Alcocer et al., 2006; Kobe et al., 2012). However, in spite of the reported age-related reduction in their brain expression levels, 5-HT_7_ receptors exert important functions also in the adult (see Section Physiological Functions Regulated by 5-HT_7_ Receptors). This conclusion is supported by several data: first of all, 5-HT_7_ receptors have been detected in the brain of adult animals (Neumaier et al., 2001; Geurts et al., 2002; Muneoka and Takigawa, 2003). In the human brain, the presence of 5-HT_7_ receptors was confirmed by post-mortem studies on adult subjects (age range 22–73 years; Martin-Cora and Pazos, 2004; Varnäs et al., 2004). Moreover, systemic administration of 5-HT_7_ receptor agonists affected body temperature (Naumenko et al., 2011) and circadian rhythms (Adriani et al., 2012; Monti et al., 2014; Romano et al., 2014) in adult animals and improved learning in young adults (Eriksson et al., 2008; Freret et al., 2014). All these data indicate that 5-HT_7_ receptor-mediated effects are still present at adult age and exert an important control on various physiological functions.

5-HT_7_ receptors display the typical structure of G protein-coupled receptors (GPCRs) with seven transmembrane domains and were initially characterized for their ability to stimulate adenylate cyclase (Bard et al., 1993; Lovenberg et al., 1993; Ruat et al., 1993; Shen et al., 1993).

Among all 5-HT receptor subtypes, 5-HT_7_ receptors display the highest affinity (in the low nanomolar range) for the natural agonist serotonin (Ruat et al., 1993). Other high affinity agonists for 5-HT_7_ receptors are 5-carboxamidotryptamine (5-CT) and 8-hydroxy-N,N-dipropyl-aminotetralin (8-OH-DPAT), both also showing high affinity for 5-HT_1A_ receptors (Bard et al., 1993; Lovenberg et al., 1993; Ruat et al., 1993). Selective agonists for 5-HT_7_ receptors were lacking until recently (Di Pilato et al., 2014). The first compound described as a 5-HT_7_ receptor agonist was AS-19, a partial agonist with high affinity but moderate selectivity for 5-HT_7_ receptors (Brenchat et al., 2009). The most selective 5-HT_7_ receptor agonists described to date are the compounds E-55888 and E-57431 (not commercially available), produced by Esteve pharmaceutical company (Brenchat et al., 2009, 2012). Another high-affinity and selective 5-HT_7_ receptor agonist is LP-211 (indicated as compound 25 in the first publication Leopoldo et al., 2008), that shows excellent brain permeation properties (Hedlund et al., 2010). *In vivo* administration of LP-211 induced hypothermia in wild-type but not in 5-HT_7_ KO mice (Hedlund et al., 2010) and shifted the sleep-wake cycle (Adriani et al., 2012), two typical 5-HT_7_ receptor-mediated effects. Other analogs of LP-211 with improved selectivity and pharmacokinetic properties have been synthesized (Leopoldo, personal communication) and are currently under investigation in our laboratories as possible novel 5-HT_7_ receptor agonists.

Concerning antagonists, several antidepressant and antipsychotic drugs showed high affinity for 5-HT_7_ receptors and behaved as antagonists on a cyclic adenosine monophosphate (cAMP) formation assay (Shen et al., 1993; Roth et al., 1994; see Section Mood Disorders). Selective and high-affinity antagonists of 5-HT_7_ receptors are also available, among which the compound SB-269970 is to date considered the most reliable (Hagan et al., 2000; Guscott et al., 2003; Hedlund et al., 2003).

### Coupling to intracellular transduction mechanisms

5-HT_7_ receptors display interesting transduction properties, being able to couple to G_s_ and G_12_ GTP-binding proteins, which activate divergent signaling pathways (reviewed by Woehler and Ponimaskin, 2009; Matthys et al., 2011; Gellynck et al., 2013).

Since their discovery, 5-HT_7_ receptors were found to be coupled to G_s_ and induce adenylate cyclase activation, cAMP formation and activation of protein kinase A (PKA; Bard et al., 1993; Lovenberg et al., 1993; Ruat et al., 1993).

Downstream to cAMP formation, 5-HT_7_ receptors can activate the extracellular signal-regulated kinase (ERK), as shown in transfected cells expressing 5-HT_7_ receptors (Lin et al., 2003; Norum et al., 2003) and in native rat hippocampal neurons (Errico et al., 2001; Lin et al., 2003). 5-HT_7_ receptor-induced activation of ERK was mediated either by PKA (Norum et al., 2003) or by a cAMP-dependent, PKA-independent pathway involving exchange proteins directly activated by cAMP (Epacs) (Lin et al., 2003). In line with this result, cAMP-dependent but PKA-independent 5-HT_7_ receptor-mediated effects have been described (Chapin and Andrade, 2001; Bonsi et al., 2007).

5-HT_7_ receptors can activate additional intracellular biochemical cascades, among which the kinase Akt (also known as protein kinase B; Hoffman and Mitchell, 2011; Johnson-Farley et al., 2005).

As mentioned above, 5-HT_7_ receptors can also couple to G_12_ (Kvachnina et al., 2005), a heterotrimeric G protein that modulates the activity of “small” monomeric GTPases (Hall, 1998), such as members of the Rho family Rho, Rac and Cdc42. Through the G12-dependent activation of RhoA and Cdc42 5-HT_7_ receptor activation regulates gene transcription and neuronal morphology (Kvachnina et al., 2005).

The mechanisms regulating 5-HT_7_ receptor coupling to G_s_ or G_12_ are not clear. Recent evidence suggests that agonist-induced dynamic palmitoylation of 5-HT_7_ receptors affects its G_s_-mediated constitutive activity with no effect on G_12_-mediated activity (Kvachnina et al., 2009; Gorinski and Ponimaskin, 2013). This result implies that pathways inducing palmitoylation of 5-HT_7_ receptors might modify their constitutive activity and switch their intracellular coupling, ultimately changing their final effect.

Another interesting finding is that the expression of G_s_ remains constant during development, whereas the expression level of G_12_ is higher at early post natal age and parallels the expression level of 5-HT_7_ receptors; consistently, 5-HT_7_ receptor-mediated effects on synapse formation and function were observed in the hippocampus of juvenile but not adult mice, indicating a crucial role of the 5-HT_7_/G_12_ pathway in the development of brain synaptic circuitry (Kobe et al., 2012).

A particular feature of 5-HT_7_ receptors, similar to other GPCRs, is the ability to form receptor complexes, either homo- or heterodimers, in which monomers reciprocally modulate receptor trafficking, ligand binding affinity and coupling to intracellular signaling cascades (Renner et al., 2012; Teitler and Klein, 2012). For example, it has been suggested that a 5-HT_1A_/5-HT_7_ heterodimer interaction plays a modulatory role in the control of body temperature (Matthys et al., 2011). This is a further element of complexity in 5-HT receptor-mediated signal transduction mechanisms.

## 5-HT_7_ receptors regulate synapse development

5-HT plays a crucial role in shaping brain structure and circuits during development through modulation of neural cell proliferation, migration and differentiation, as well as neurite outgrowth, axonal guidance and synaptogenesis (Gaspar et al., 2003). Accordingly, early changes in 5-HT brain levels during development affect the functional organization of brain networks and may underlie the pathogenesis of neurodevelopmental disorders including autism (reviewed by Lesch and Waider, 2012). While the role of some 5-HT receptors during brain development has been ascertained (Sodhi and Sanders-Bush, 2004), the involvement of 5-HT_7_ receptors has only recently emerged. As mentioned above, the group of Ponimaskin demonstrated that in mouse hippocampal neurons 5-HT_7_ receptor activation stimulated the small GTP-ases RhoA and Cdc42 and enhanced neurite elongation, dendritic spine density, the number of synaptic contacts and the amount of AMPA receptors expressed at synapses, leading to increased synaptic efficacy (Kvachnina et al., 2005; Kobe et al., 2012). In line with these results, it was recently shown that the 5-HT_7_ receptor agonists 8-OH-DPAT and LP-211 stimulated neurite outgrowth in primary cultures of mouse and rat striatal and cortical neurons by activation of the cyclin-dependent protein kinase 5 (Cdk5), a kinase playing an important role in microtubule assembly and cytoarchitecture rearrangements (Speranza et al., 2013).

## Modulation of neuronal excitability by 5-HT_7_ receptors

### 5-HT_7_ receptor activation exerts depolarizing effects

**Figure 1:**
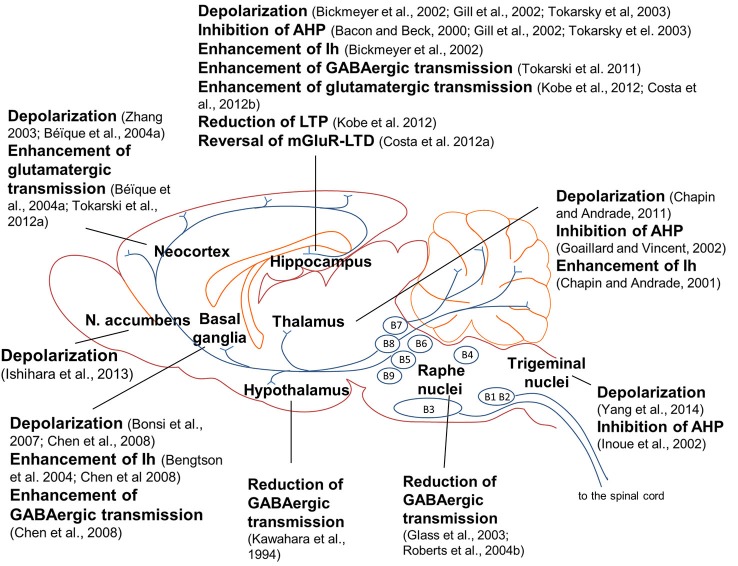
**Effects of 5-HT_7_ receptor activation on neuronal excitability, synaptic transmission and synaptic plasticity in the rodent brain**. Activation of 5-HT_7_ receptors induced depolarization in several brain regions; in many cases, depolarization was mediated by inhibition of a post-spike afterhyperpolarization (AHP) and/or enhancement of a hyperpolatization-activated cation current (Ih; see explanations in the text). Activation of 5-HT_7_ receptors modulated glutamate-mediated synaptic transmission and plasticity in frontal cortex and hippocampus. 5-HT_7_ receptors differently modulated GABAergic synaptic transmission in distinct brain areas; notably, activation of 5-HT_7_ receptors in raphe nuclei inhibited GABAergic interneurons, enhancing the activity of serotonergic raphe neurons and 5-HT release in target structures. Most of the results illustrated here were obtained in the rat, with the following exceptions: mouse (Bickmeyer et al., 2002; Costa et al., 2012a; Yang et al., 2014), hamster (Glass et al., 2003) and guinea pig (Roberts et al., 2004b).

Brain 5-HT_7_ receptors modulate neuronal networks by playing a crucial role in brain wiring during development. However, as mentioned before, 5-HT_7_ receptor-mediated effects are not restricted to a defined developmental window, as a large number of studies show that 5-HT_7_ receptors modulate neuronal excitability, synaptic transmission and synaptic plasticity both during development and adult life. 5-HT_7_ receptors control neuronal intrinsic excitability by modulating non-synaptic membrane ion currents which directly affect neuronal firing. 5-HT_7_ receptor agonists exerted slow depolarizing effects and increased neuronal firing in several brain areas (Figure 1), among which the anterodorsal nucleus of the rat thalamus (Chapin and Andrade, 2001), rat globus pallidus (Chen et al., 2008), mouse hippocampal CA1 region (Bickmeyer et al., 2002) and mouse trigeminal nucleus caudalis (Yang et al., 2014). Activation of 5-HT_7_ receptors enhanced the firing rate of rat CA1 pyramidal neurons (Tokarski et al., 2003), CA3 pyramidal neurons (Gill et al., 2002), striatal cholinergic interneurons (Bonsi et al., 2007), prefrontal cortex pyramidal neurons (Zhang, 2003; Béïque et al., 2004a) and nucleus accumbens (NAc) neurons (Ishihara et al., 2013). In a spinal cord slice preparation, 5-HT application induced a locomotor-like rhythmic firing activity of motoneurons in wild type but not in 5-HT_7_ KO mice, indicating that the excitability of spinal motoneurons was enhanced by 5-HT_7_ receptors (Liu et al., 2009).

In many of the studies above cited, 5-HT_7_ receptor-induced depolarization was mediated by inhibition of a post-spike AHP or by enhancement of a hyperpolarization-activated inward cation current (Ih).

### 5-HT_7_ receptor activation inhibits post-spike afterhyperpolarization (AHP)

Action potentials are followed by a negative shift in membrane potential named afterhyperpolarization (AHP), which is mediated by a Ca^2+^-dependent K^+^ current triggered by Ca^2+^ influx. The subsequent efflux of K^+^ ions hyperpolarizes the membrane during a few seconds reducing neuronal firing rate, a phenomenon called “spike frequency adaptation” (Sah, 1996). Activation of 5-HT_7_ receptors inhibited a slow AHP (sAHP) in thalamic neurons (Goaillard and Vincent, 2002), in CA3 pyramidal neurons (Bacon and Beck, 2000; Gill et al., 2002) and in CA1 pyramidal neurons (Tokarski et al., 2003; Figure 1). Likewise, in rat trigeminal motoneurones 5-HT_7_ receptor activation reduced a Ca^2+^-dependent K^+^ current responsible for a medium-duration afterhyperpolarization (mAHP), another type of AHP with a time-course faster than the sAHP and with similar inhibitory effects on neuronal excitability (Inoue et al., 2002). Modulation of the AHP by 5-HT_7_ receptors was mediated by activation of the cAMP/PKA pathway (Goaillard and Vincent, 2002; Inoue et al., 2002).

Modulation of the AHP has profound effects on neuronal firing. By inhibiting post-spike mAHP and sAHP, 5-HT_7_ receptor activation enhances neuronal firing rate and reduces spike frequency adaptation. In addition, 5-HT_7_ receptor-mediated modulation of sAHP is likely to involve broader functional consequences, as intrinsic excitability and synaptic inputs mutually influence each other. In fact, Ca^2+^ influx through N-methyl-D-aspartate (NMDA) channels can also activate the sAHP (Lancaster et al., 2001), which implies that synaptic activation also modulates the AHP. Changes in AHP amplitude have been observed following activation of NMDA and kainate receptors (Cherubini et al., 1990) and group I metabotropic glutamate receptors (mGluRs; Ireland and Abraham, 2002).

The AHP in turn reduces NMDA-mediated synaptic transmission: it has been proposed that activation of AHP, by hyperpolarizing the membrane, would favor Mg^2+^ blockade of NMDA channels and reduce NMDA-mediated transmission and plasticity (Wu et al., 2004; Fernández de Sevilla et al., 2007). Vice-versa, conditions reducing the AHP lead to depolarization and removal of Mg^2+^ blockade, thus are likely to enhance NMDA-mediated long term plasticity and learning. Consistent with this hypothesis, it was shown that the amplitude of AHP in hippocampal neurons was decreased after learning (Disterhoft et al., 1996; Moyer et al., 1996; Oh et al., 2009) and age-related learning deficits are accompanied by increased AHP (Disterhoft et al., 1996; Tombaugh et al., 2005).

Since 5-HT_7_ receptor activation is able to inhibit post-spike AHP in many brain regions, as described above, the subsequent depolarization might favor NMDA receptor-mediated synaptic plasticity and ultimately enhance learning. As a matter of fact, several studies indicate that 5-HT_7_ receptor activation exerts pro-cognitive effects (see Section Learning and Memory).

### 5-HT_7_ receptor activation enhances a hyperpolarization-activated cation current (I_h_)

Another non-synaptic ion current modulated by 5-HT_7_ receptors is a hyperpolarization-activated cation current (I_h_). I_h_ is carried by Na^+^ and K^+^ ions and is activated by hyperpolarization beyond −60 mV, thus is already active at resting membrane potential. The voltage-dependence of I_h_ activation is modulated by cAMP, with increases in cAMP levels facilitating I_h_ activation (Chen et al., 2001). The physiological consequences of I_h_ activation are very complex, as it differently affects membrane excitability and synaptic responsiveness. Concerning membrane excitability, I_h_ shifts the value of resting membrane potential towards depolarization. With respect to synaptic responsiveness, I_h_ instead exerts a membrane-shunting effect that reduces the amplitude and duration of excitatory post-synaptic potentials. Thus, an enhancement of I_h_, although producing a depolarizing shift in membrane potential, ultimately decreases synaptic responsiveness, as it reduces neuronal ability to elicit action potentials in response to synaptic inputs. I_h_ also reduces the temporal summation of synaptic signals. Notably, the channels responsible for I_h_ (hyperpolarization-activated cyclic nucleotide-gated non-specific cation channels, HCN) are maximally expressed in distal dendrites, where integration of synaptic signals mostly occurs (Magee, 1998, 1999); reviewed by Mozzachiodi and Byrne (2010).

It was shown that 5-HT_7_ receptor activation enhanced I_h_ in dorsal root ganglion neurons (Cardenas et al., 1999), in mouse hippocampus (Bickmeyer et al., 2002), in the anterodorsal thalamic nucleus (Chapin and Andrade, 2001) and in rat globus pallidus (Bengtson et al., 2004; Chen et al., 2008; Figure 1). In most cases, 5-HT_7_ receptor-mediated enhancement of I_h_ was mediated by an increase in cAMP levels (Cardenas et al., 1999; Chapin and Andrade, 2001; Bickmeyer et al., 2002; Bengtson et al., 2004).

Neurotransmitter-mediated modulation of I_h_ has important functional consequences on neuronal rhythmic firing (McCormick and Pape, 1990). I_h_ is also suggested to be involved in epilepsy, since either an increase (Surges et al., 2012) or a decrease (Jung et al., 2007, 2011) of I_h_ have been reported in different types of epilepsy.

Changes in I_h_ also occur during long-term synaptic plasticity (reviewed by Mozzachiodi and Byrne, 2010). I_h_ was increased during NMDA-mediated long term potentiation (LTP; Fan et al., 2005) and decreased after the induction of metabotropic glutamate receptor-mediated long term depression (mGluR-LTD; Brager and Johnston, 2007). Interestingly, it was recently shown that hippocampal I_h_ is altered in Fmr1KO mice, the mouse model of Fragile X syndrome, in which mGluR-mediated signaling is deregulated. In Fmr1 KO mice, I_h_ is abnormally enhanced in the apical dendrites but not in the soma of CA1 pyramidal neurons and NMDA-mediated modulation of I_h_ is disrupted (Brager et al., 2012; Brager and Johnston, 2014). The authors suggested that the subsequent reduction in dendritic synaptic integration together with enhanced intrinsic excitability may participate to epilepsy, a typical feature of Fmr1KO mice as well as of Fragile X patients.

In summary, modulation of I_h_ by 5-HT_7_ receptors is likely to have important consequences on neuronal firing and synaptic responsiveness and might account for the effects exerted by 5-HT_7_ receptor activation on synaptic transmission and on epilepsy (see below).

## Modulatory role of 5-HT_7_ receptors on synaptic transmission and plasticity

### Effects of 5-HT_7_ receptor activation on GABAergic synaptic transmission

5-HT_7_ receptors differently modulate the activity of GABAergic inhibitory interneurons in distinct brain areas (Figure 1). An early study showed that 5-HT_7_ receptor activation reduced GABA_A_ receptor-activated current in cultured rat SCN neurons acting through a post-synaptic cAMP-mediated mechanism (Kawahara et al., 1994). Also in raphe nuclei 5-HT_7_ receptor activation reduced GABAergic transmission. In raphe nuclei, GABAergic interneurons exert a negative control on the activity of serotonergic neurons, inhibiting 5-HT release from raphe efferent fibers. When a 5-HT_7_ receptor antagonist was applied in raphe nuclei, 5-HT efflux onto target structures was reduced (Glass et al., 2003; Roberts et al., 2004b). Thus, activation of 5-HT_7_ receptors in raphe nuclei reduces GABA-mediated inhibition of raphe serotonergic neurons and consequently enhances 5-HT release in target structures.

In the hippocampus, 5-HT_7_ receptor activation was instead shown to stimulate the activity of GABAergic interneurons. Application of a 5-HT_7_ receptor agonist enhanced the frequency of GABA-mediated spontaneous inhibitory post-synaptic currents (sIPSCs) recorded from rat CA1 pyramidal neurons, indicating an increased GABA release from interneurons (Tokarski et al., 2011). The authors suggest that 5-HT_7_ receptors exerted two effects, both at a pre-synaptic level: an enhancement of glutamate release from fibers targeting GABAergic interneurons and an increase of GABA release from interneuron terminals. In the CA1 region, GABAergic interneurons represent a system of feed-forward and feedback inhibition of pyramidal neurons, being activated by afferent fibers (among which the raphe-hippocampal serotonergic pathway), by Schaffer collaterals as well as by recurrent collaterals from pyramidal neurons (Freund and Buzsaki, 1996). Therefore, 5-HT_7_ receptors exert a very complex modulation of hippocampal circuits, as they directly depolarize pyramidal neurons (Tokarski et al., 2003) and simultaneously regulate their firing by enhancing GABAergic inhibitory control.

5-HT_7_ receptor activation enhanced GABAergic transmission also in rat globus pallidus (Chen et al., 2008). To summarize, in the hippocampus and globus pallidus 5-HT_7_ receptor activation stimulates GABA release from interneurons, differing from results observed in suprachiasmatic nucleus (SCN) and dorsal raphe, where 5-HT_7_ receptor activation instead reduces GABAergic transmission.

### Effects of 5-HT_7_ receptor activation on glutamate-mediated synaptic transmission and plasticity

Two different reports indicate that 5-HT_7_ receptor activation can stimulate glutamate release from glutamatergic terminals. In rat frontal cortex, glutamate-mediated synaptic transmission was enhanced by activation of 5-HT_7_ receptors (Béïque et al., 2004b) and decreased by the 5-HT_7_ receptor antagonist SB-269970 (Tokarski et al., 2012a; Figure 1). In both studies, 5-HT_7_ receptor-mediated effect was exerted at a pre-synaptic level on glutamatergic terminals.

In the hippocampus, 5-HT_7_ receptors modulate glutamate-mediated transmission acting at a post-synaptic level. Field recordings of excitatory post-synaptic potentials (EPSPs) from rat hippocampal slices have shown that serotonin inhibits the perforant path input to the CA1 region, an effect partially mediated by 5-HT_7_ receptors and exerted at a post-synaptic level (Otmakhova et al., 2005). The authors suggest that 5-HT_7_ receptor-mediated reduction of EPSP amplitude might be due to an enhancement of the hyperpolarization-activated current I_h_, similar to what observed in several brain regions (see above).

The other main input to CA1 pyramidal neurons is represented by Schaffer collaterals from CA3 pyramidal neurons. We have studied the effects of serotonin on glutamatergic transmission in the CA3-CA1 synapse in mouse and rat hippocampal slices and showed that activation of post-synaptic 5-HT_7_ receptors enhances the amplitude of the AMPA receptor-mediated component of the excitatory post synaptic current (EPSC_AMPA_), which is responsible for basal glutamatergic transmission (Costa et al., 2012b). In the same study, we observed instead that 5-HT_1A_ receptors inhibit AMPA-mediated CA3-CA1 synaptic transmission. These data provide a physiological substrate to the previous observation that 5-HT_7_ receptor activation exerts a pro-cognitive action and is able to counteract 5-HT_1A_-mediated impairment of learning (Eriksson et al., 2008).

In the last few years, evidence has emerged that 5-HT_7_ receptors can also modulate long-term synaptic plasticity (Figure 1). As already mentioned, in mice hippocampal neurons activation of 5-HT_7_ receptors enhanced basal synaptic transmission by increasing the number of AMPA receptors at synapses but also modulates glutamate-mediated long-term synaptic plasticity, reducing the amount of LTP in the CA3-CA1 synapse (Kobe et al., 2012). The authors suggested that 5-HT_7_ receptor-mediated enhancement of basal glutamatergic transmission might have prevented further potentiation, thus reducing LTP (Kobe et al., 2012). In contrast with this report, mice lacking 5-HT_7_ receptors (5-HT_7_ KO) display decreased LTP in the CA3-CA1 hippocampal synapse (Roberts et al., 2004a), suggesting that 5-HT_7_ receptors are necessary for LTP. LTP is induced by activation of NMDA receptors and 5-HT_7_ receptors were shown to exert different short- and long-term effects on NMDA-mediated currents in hippocampal neurons. In particular, acute activation of 5-HT_7_ receptors enhanced the amplitude of NMDA-mediated currents, whereas a long-term activation of 5-HT_7_ receptors reduced the cell membrane expression of NMDA receptors through an indirect mechanism involving platelet-derived growth factor (PDGF), an endogenous neurotrophin that protects against NMDA-induced excitotoxicity (Vasefi et al., 2013). This dual action of 5-HT_7_ receptors, namely a short-term enhancement and a long-term inhibition of NMDA receptors, might also explain different 5-HT_7_ receptor-mediated effects on LTP.

Another form of long-term depression, mGluR-LTD, is induced by activation of group I mGluRs and is mainly expressed through removal of AMPA receptors from synaptic membrane surface by endocytosis (Luscher and Huber, 2010). We have shown that 5-HT_7_ receptor activation prevented mGluR-induced endocytosis of AMPA receptors and reversed mGluR-LTD in the CA3-CA1 synapse in mouse hippocampal slices (Costa et al., 2012a). Interestingly, we found that 5-HT_7_ receptor activation reversed mGluR-mediated endocytosis of AMPA receptors and mGluR-LTD also in Fmr1 KO mice, a mouse model of Fragile X Syndrome, the most common form of inherited intellectual disability associated with epilepsy and autism. Our result that 5-HT_7_ receptor activation can correct abnormal mGluR-mediated synaptic plasticity in the mouse model of Fragile X Syndrome might suggest new strategies for the therapy of this disorder (see below).

## Physiological functions regulated by 5-HT_7_ receptors

5-HT_7_ receptors control several body functions including the sleep-wake cycle, body temperature and nociception, and strongly influence mood and learning, two higher brain functions that are strictly related (Table 1). Most of the functions regulated by 5-HT_7_ receptors have been studied using the 5-HT_7_ receptor knock-out (5-HT_7_ KO) mice that were generated and characterized by the research group of Dr. Hedlund (Roberts et al., 2004a; Sarkisyan and Hedlund, 2009) and later in other laboratories (Guscott et al., 2005; Witkin et al., 2007).

### Sleep/wake cycle

Consistent with their abundant expression in the hypothalamus (see Section Localization, Structure and Pharmacological Profile), 5-HT_7_ receptors play an important role in the regulation of circadian rhythm and sleep (Monti and Jantos, 2014). In the SCN, which is the main regulator of circadian rhythm, 5-HT_7_ receptors are abundantly expressed (Belenky and Pickard, 2001) and regulate glutamatergic input from the retina (Smith et al., 2001) and serotonergic input from raphe nuclei (Glass et al., 2003).

The role of 5-HT_7_ receptors in sleep has emerged from studies using both pharmacological and genetic approaches. 5-HT_7_ KO mice displayed a reduced duration of REM sleep, whereas time spent during wake or non-REM sleep was not different from wild-type mice (Hedlund et al., 2005). Consistently, *in vivo* administration of a 5-HT_7_ receptor antagonist to wild-type animals reduced the duration of REM sleep (Hagan et al., 2000; Thomas et al., 2003; Bonaventure et al., 2007; Monti et al., 2012). However, in contradiction with these results, systemic administration of a 5-HT_7_ receptor agonist also enhanced the wake period and reduced the duration of REM sleep (Monti et al., 2008, 2014), together with inducing a phase-shift in the sleep-wake cycle (Adriani et al., 2012). As a possible explanation, it has been proposes that 5-HT_7_ receptors do not behave according to the classical model of two-state on-off (activation/blockade) ligand-receptor interaction (Monti and Jantos, 2014). In addition, 5-HT_7_ receptors differently modulate distinct brain areas (dorsal raphe, locus coeruleus, basal forebrain) involved in sleep control (Monti et al., 2012; Monti and Jantos, 2014) and their precise role in each area should be further investigated.

### Body temperature

5-HT_7_ receptors exert a well-established role in thermoregulation (for extensive review on this topic, see Hedlund and Sutcliffe, 2004; Matthys et al., 2011). Administration of 5-HT_7_ receptor agonists induced hypothermia in wild type guinea pigs and mice (Hagan et al., 2000; Guscott et al., 2003; Hedlund et al., 2003, 2010) but not in 5-HT_7_ KO mice (Guscott et al., 2003; Hedlund et al., 2003).

Two other 5-HT receptors are involved on thermoregulation, namely 5-HT_1A_ and 5-HT_3_ receptors, both also inducing hypothermic effects (Hedlund et al., 2004; Naumenko et al., 2011). It has been proposed that 5-HT_7_ receptors, possessing the highest affinity for 5-HT, are activated by low doses of 5-HT and are responsible for the fine-tuning of body temperature. On the other hand, higher agonist concentrations activate 5-HT_1A_ receptors, which might play a role as a defense against hyperthermia (Hedlund et al., 2004).

### Nociception

Immunohistochemical studies provide evidence for localization of 5-HT_7_ receptors in the superficial laminae of the dorsal horn and in small and medium sized dorsal root ganglion cells, which is consistent with a role of 5-HT_7_ receptors in nociception (Doly et al., 2005).

The role of 5-HT_7_ receptors in the control of pain transmission is multifaceted and has been described in details in recent reviews (Matthys et al., 2011; Viguier et al., 2013). Overall, 5-HT_7_ receptor agonists induce pro-nociceptive effects on peripheral 5-HT_7_ receptors and antinociceptive effects on central 5-HT_7_ receptors (Yanarates et al., 2010; Viguier et al., 2012), with a different outcome depending on preexisting conditions (health vs. neuropathic pain; Viguier et al., 2013).

### Learning and memory

In the last decade, an important role of 5-HT_7_ receptors on learning and memory has emerged (Roberts and Hedlund, 2012; Meneses, 2013). As pointed out in the previous paragraphs, 5-HT_7_ receptors are expressed at high levels in the hippocampus, one of the brain regions most crucially involved in learning, and modulate hippocampal synaptic transmission and plasticity. Behavioral studies performed on mice lacking 5-HT_7_ receptors (Roberts et al., 2004a; Sarkisyan and Hedlund, 2009) and on wild-type animals (Manuel-Apolinar and Meneses, 2004; Perez-Garcia and Meneses, 2005; Eriksson et al., 2008, 2012) show that 5-HT_7_ receptor activation exerts a pro-cognitive action on different types of memory.

5-HT_7_ KO mice showed no memory impairment in operant food conditioning tests (involving a hippocampus-independent type of memory) and Barnes maze (involving hippocampus-dependent spatial learning) but displayed a memory deficit in the fear conditioning test, which involves hippocampus-dependent contextual learning with emotional aspects. These results indicate that the lack of 5-HT_7_ receptors did not affect hippocampus-independent memory and among different types of hippocampus-dependent learning, only one with a strong emotional component (fear conditioning) was selectively impaired in 5-HT_7_ KO mice. The specific contextual learning impairment of 5-HT_7_ KO mice was accompanied by a decrease of LTP in the CA1 region of the hippocampus (Roberts et al., 2004a).

Further studies from the same laboratory indicated that 5-HT_7_ KO mice displayed normal recognition of novel objects (Sarkisyan and Hedlund, 2009), a type of visual episodic memory that depends on brain cortex and is believed to correspond to human declarative episodic memory. 5-HT_7_ KO mice instead showed a selective impairment in the recognition of a novel location, particularly in allocentric spatial memory (a hippocampus-dependent type of memory concerning the location of objects independent from the observer), without any defect in egocentric memory (a striatum-dependent type of memory concerning the position of objects with respect to the observer). The same result was observed in wild-type mice following pharmacological blockade of 5-HT_7_ receptors (Sarkisyan and Hedlund, 2009).

Behavioral studies on wild-type animals confirmed that activation of 5-HT_7_ receptors exerts a pro-cognitive effect on hippocampus-dependent contextual learning and revealed that other types of memory are also modulated by 5-HT_7_ receptors. In mice submitted to the passive avoidance test (involving contextual learning), *in vivo* activation of 5-HT_7_ receptors was able to exert pro-cognitive effects and counteract a learning impairment induced by 5-HT_1A_ receptor activation (Eriksson et al., 2008, 2012).

5-HT_7_ receptor stimulation enhanced memory formation in adult rats trained to a learning task involving both conditioning (Pavlonian) and instrumental learning modes: in this experimental protocol, a food reward was delivered with a short delay following a light signal (conditioned learning) but was delivered immediately if the rat pressed a lever (instrumental learning). When the 5-HT_7_ receptor agonist AS-19 was injected subcutaneously to animals immediately after training, the latency of responses was reduced, indicating enhanced memory performance. AS-19 administration was also able to reverse a memory impairment induced by scopolamine (an anticholinergic agent) or dizocilpine (an NMDA antagonist), suggesting that 5-HT_7_ receptor activation might exert a pro-cognitive effect by modulating cholinergic and glutamatergic transmission (Perez-Garcia and Meneses, 2005).

Concerning hippocampus-independent memory, different from results on 5-HT_7_ KO mice (Sarkisyan and Hedlund, 2009), in wild-type mice novel object recognition was enhanced by administration of the 5-HT_7_ receptor agonist 5-CT and impaired by administration of the 5-HT_7_ receptor antagonist SB-269970 (Freret et al., 2014), consistent with previous data showing that administration of SB-269970 impaired novel object recognition in wild-type rats (Ballaz et al., 2007a). These data show that 5-HT_7_ receptor activation exert a pro-cognitive effect also on cortex-dependent memory corresponding to human episodic memory.

To summarize, studies on wild-type animals using 5-HT_7_ receptor agonists and antagonists have shown that 5-HT_7_ receptor activation enhances many different types of memory, including some (operant conditioning, novel object recognition) that were not impaired in 5-HT_7_ KO mice.

5-HT_7_ receptor-mediated pro-cognitive effects were observed in behavioral tests performed on adult animals, reinforcing the conclusion that 5-HT_7_ receptors are functional in the adult hippocampus and exert life-long effects on learning and memory.

In one study, blockade rather than activation of 5-HT_7_ receptors exerted a pro-cognitive effect: *in vivo* administration of the 5-HT_7_ receptor antagonist SB-269970 to rats submitted to the radial arm maze enhanced long-term reference memory without affecting short-term working memory (Gasbarri et al., 2008).

Heterogeneous results are not surprising considering that different models have been used (either rats or mice; wild-type or 5-HT_7_ KO animals) and different types of memory were tested, involving different brain areas and distinct circuits. Furthermore, considering that learning and memory are strictly related to stress, 5-HT_7_ receptor-mediated effects on learning might also be influenced by 5-HT_7_ receptor-mediated effects on mood (see below).

## Involvement of 5-HT_7_ receptors in pathology

### Mood disorders

Since the first pharmacological characterization of 5-HT_7_ receptors, it was evident that several antipsychotics and antidepressants bind these receptors with high affinity (Monsma et al., 1993; Roth et al., 1994). In line with these early results, several studies have later shown that 5-HT_7_ receptors play a role in mood regulation and thus are potential targets for the therapy of anxiety and depression (Hedlund, 2009; Sarkisyan et al., 2010).

Mice lacking 5-HT_7_ receptors displayed reduced levels of depression when submitted to forced swimming and tail suspension tests (Guscott et al., 2005; Hedlund et al., 2005). Consistently, pharmacological blockade of 5-HT_7_ receptors exerted antidepressant effects in wild-type mice and rats submitted to the same stress protocols (Hedlund et al., 2005; Wesolowska et al., 2006a; Mnie-Filali et al., 2011). In addition, 5-HT_7_ receptor blockade was found to potentiate the effect of other antidepressant drugs: synergistic antidepressant effects were observed when a low and ineffective dose of SB-269970 was administered in combination with a low and ineffective dose of citalopram, a selective serotonin reuptake inhibitor (SSRI; Sarkisyan et al., 2010). In the same study, a synergistic interaction, although to a lesser extent, was also observed between SB-269970 and reuptake inhibitors of norepinephrine (but not dopamine).

With respect to anxiety, 5-HT_7_ KO mice did not show any alteration in anxiety-related behavior, evaluated by light-dark transfer test (Roberts et al., 2004a) and elevated plus maze (Guscott et al., 2005). On the other side, in wild-type rodents tested in different anxiety protocols (Vogel conflict drinking test; elevated plus maze; four-plate test; open field), administration of the 5-HT_7_ receptor antagonist SB-269970 induced anxiolytic effects (Wesolowska et al., 2006a,b).

Interestingly, anxiolytic and antidepressant effects were observed also when SB-269970 was administered by local intra-hippocampal injection, indicating the involvement of 5-HT_7_ receptors located in the hippocampus (Wesolowska et al., 2006a).

It was recently found that second-generation antipsychotic drugs, named atypical antipsychotics, also behave as antagonists of 5-HT_7_ receptors. Among these substances, the D2/D3 receptor antagonist amisulpride is a high-affinity competitive antagonist of 5-HT_7_ receptors. Interestingly, the antidepressant effect of amisulpride was absent in 5-HT_7_ KO mice, indicating that it was mediated exclusively by inhibition of 5-HT_7_ receptors (Abbas et al., 2009). Lurasidone, another atypical antipsychotic behaving as an antagonist at dopamine D2, 5-HT_2_, and 5-HT_7_ receptors (Ishibashi et al., 2010), was shown to exert significant antidepressant effects in patients with bipolar 1 disorder (Woo et al., 2013). Another atypical antipsychotic is vortioxetine, also named Lu AA21004, a multimodal compound that behaves as an antagonist at 5-HT_3_ and 5-HT_7_ receptors, a partial agonist at 5-HT_1B_, a full agonist at 5-HT_1A_ receptors and an inhibitor of the serotonin transporter (Guilloux et al., 2013). Vortioxetine proved effective as antidepressant in several clinical studies and was recently approved by the U.S. Food and Drug Administration (FDA) for the treatment of major depressive disorder (Citrome, 2014), confirming that an enhancement of serotonergic transmission associated with 5-HT_7_ receptor antagonism exerts synergistic antidepressant effects.

These data together indicate that 5-HT_7_ receptors regulate the balance between anxiety and depression. Mood regulation has crucial consequences on cognition, as stress can improve or impair learning depending on the duration and intensity of stressful conditions. Acute stress enhances learning, particularly when experienced in the same context of learning acquisition (Joëls et al., 2006; Bangasser and Shors, 2010). As underlined above, 5-HT_7_ receptor activation improves fear-related learning, in which an acute stressor is presented during the paradigms of fear conditioning and passive avoidance (Roberts et al., 2004a; Eriksson et al., 2008). Therefore, an enhancement of anxiety levels by 5-HT_7_ receptor activation during a stress condition might contribute to 5-HT_7_ receptor-mediated improvement of stress-related learning.

### Epilepsy

In view of the depolarizing effects exerted by 5-HT_7_ receptors (see above), their activation is likely to enhance seizure sensitivity. In line with this, a pharmacological study using different mixed antagonists suggested that blockade of 5-HT_7_ receptors may protect against audiogenic seizures in DBA/2J mice (Bourson et al., 1997). Pharmacological blockade using selective 5-HT_7_ receptor antagonists decreased cortical epileptic activity in a rat genetic model of absence epilepsy (Graf et al., 2004) and in a pilocarpine-induced rat model of temporal lobe epilepsy (Yang et al., 2012).

In contrast, 5-HT_7_ receptor activation was instead suggested to exert anticonvulsant effects in other experimental models of epilepsy. Systemic administration of the 5-HT_7_ receptor agonist 5-CT reduced picrotoxin-induced seizures in mice, an effect abolished by a selective 5-HT_7_ receptor antagonist (Pericic and Svob Strac, 2007). Consistently, mice lacking 5-HT_7_ receptors display enhanced sensitivity to electrically and chemically-induced seizures (Witkin et al., 2007).

Thus, 5-HT_7_ receptor-mediated effects on epilepsy are heterogeneous and apparently controversial, probably due to differences in the models used. Systemic administration of 5-HT_7_ receptor ligands exerts a complex modulation of the brain serotonin system because, besides directly acting on 5-HT_7_ receptors on target cells, they also modulate the activity of GABAergic interneurons. As pointed out in the previous paragraphs, in raphe nuclei 5-HT_7_ receptor activation inhibits GABAergic interneurons targeting serotonergic neurons and subsequently enhance 5-HT release in other brain areas. In the hippocampus, GABAergic interneurons are instead stimulated by 5-HT_7_ receptor activation. Therefore, the understanding of systemic effects exerted by 5-HT_7_ receptor ligands is complicated by 5-HT_7_ receptor-mediated modulation of GABAergic interneurons in distinct brain areas.

Concerning systemic administration of 5-HT_7_ receptor agonists, it should also be considered that 5-HT_7_ receptors undergo desensitization following prolonged activation (Shimizu et al., 1998). Desensitization of 5-HT_7_ receptors was also observed after antagonist treatment (Tokarski et al., 2012b). Thus, systemic treatments of animals should be carefully designed to minimize 5-HT_7_ receptor desensitization; in this respect, a preferential use of agonists with a short half-life, such as LP-211 and related compounds (Hedlund et al., 2010) might be considered preferentially.

## The serotonin hypothesis of autism

A large body of evidence has led to the serotonin hypothesis of autism, which points out a deficiency in the brain serotonin system as a causal mechanism in ASD (Whitaker-Azmitia, 2005; Harrington et al., 2013). Early experimental data (Schain and Freedman, 1961), later confirmed by many research groups (Anderson et al., 1990; Piven et al., 1991; McBride et al., 1998; Mulder et al., 2004), have documented an increase of serotonin levels in blood platelets (hyperserotonemia) in one third of autistic patients. Conversely, a decreased uptake of tryptophan (the precursor of 5-HT) and a reduced 5-HT synthesis were detected in the brain of autistic children by positron emission tomography (PET) using the radioligand tracer alpha-methyl-tryptophan (Chugani et al., 1997, 1999; Chandana et al., 2005). These studies have evidenced that global brain 5-HT synthesis was reduced in autistic patients in an age-dependent manner, with local differences in cortical regions. The localization of 5-HT defect was related to the severity of language problems, as children with the most severe language delay displayed the lowest amount of tryptophan uptake in the left cortex. Overall, in very young autistic children (2–5 years old) the rate of 5-HT synthesis in frontal, temporal and parietal cortex was significantly lower with respect to healthy children of the same age and was gradually increased only later in development (5–14 years old). The authors suggested that a high brain serotonin synthesis normally occurring during early childhood is disrupted in autism (Chugani et al., 1999).

A lack of 5-HT during early stages of development is likely to disrupt the wiring architecture of the brain. Consistently, post mortem observation of brains from young autistic patients showed an abnormal morphology of serotonergic fibers directed to the amygdala and temporal cortex and an increased density of the serotonin transporter (named either 5-HTT or SERT) on these fibers (Azmitia et al., 2011).

The 5-HTT is responsible for 5-HT uptake in CNS serotonergic nerve terminals, reducing the amount of 5-HT in the synaptic cleft. The 5-HTT is also located on blood platelets and up-regulation of 5-HTT activity participates to their increased uptake of serotonin, leading to hyperserotonemia (Marazziti et al., 2000). The gene coding for the 5-HTT, named SLC6A4, exists in various alleles related to different degrees of 5-HTT expression and/or activity. Polymorphism of the SLC6A4 gene has been correlated with autism, although results from different groups are heterogeneous with respect to the polymorphic sites involved and the type of allele associated with autism (Devlin et al., 2005; Cho et al., 2007; Coutinho et al., 2007; Wassink et al., 2007; Cross et al., 2008); however, others did not find any significant association (Ramoz et al., 2006). A SLC6A4 variant coding for an overactive form of 5-HTT has been identified in families of autistic patients (Sutcliffe et al., 2005). Mutant mice expressing this high functioning 5-HTT variant show hyperserotonemia, hypersensitivity of 5-HT receptors (as a result of reduced serotonergic transmission) and autistic behavior (Veenstra-VanderWeele et al., 2012).

Alterations in brain 5-HT receptor density were found in autistic patients. A reduction of 5-HT_2A_ binding sites in the cingulate, frontal, and temporal cortex was detected in adults with Asperger syndrome by single photon emission computed tomography (SPECT; Murphy et al., 2006), although a later PET study found contrasting results (Girgis et al., 2011). A reduced density of 5-HT_1A_ and 5-HT_2_ receptors in posterior cingulate cortex and fusiform cortex, brain regions involved in social and emotional behaviors, was observed in post-mortem brain tissue from young adults diagnosed with autism other than Asperger syndrome (Oblak et al., 2013).

To summarize, a large number of clinical studies indicate abnormal synthesis and increased uptake of 5-HT, morphological alteration of serotonergic fibers and reduced expression of 5-HT receptors in the brain of ASD patients. Consistently, neonatal mice depleted of forebrain serotonin by neurotoxin injection at birth (Boylan et al., 2007) and mice genetically depleted of serotonin (Kane et al., 2012) show a delay in development and typical autistic features, and have been proposed as animal models of ASD. Other animal models of ASD were instead generated by manipulations inducing pre-natal hyperserotonemia and postnatal loss of brain 5-HT, such as fetal exposure to sodium valproate (Dufour-Rainfray et al., 2011) or to 5-methoxytryptamine (5-MT), a non-selective agonist of all metabotropic 5-HT receptor types (McNamara et al., 2008). According to a developmental theory of autism, prenatal hyperserotonemia causes increased 5-HT levels in the fetal brain, as the immature blood-brain barrier is permeable to 5-HT; this in turn would cause a loss of serotonergic fibers as a negative feedback mechanism (Whitaker-Azmitia, 2005; McNamara et al., 2008). Thus, prenatal hyperserotonemia would ultimately reduce the function of the brain serotonin system and lead to consequences similar to post-natal serotonin depletion.

Animal studies have confirmed the involvement of 5-HT in ASD and provided some indication about the 5-HT-dependent mechanisms disrupted in ASD. In 5-HTT knockout animals, the lack of 5-HTT during early development altered the connectivity between raphe nuclei and prefrontal cortex (Witteveen et al., 2013), as well as cortical cell density and layer thickness (Altamura et al., 2007; Witteveen et al., 2013). Also neonatal 5-HT depletion in mouse forebrain caused a disruption in neocortical architecture, namely an increase of cortical thickness (Boylan et al., 2007) resembling the increased cortical volume observed in autistic patients (Carper and Courchesne, 2005).

In a hyperserotonemia rat model of autism, a reduced number of oxytocin neurons was detected in the hypothalamic paraventricular nucleus (PVN; McNamara et al., 2008). The PVN projects oxytocin-containing fibers to the NAc, where pre-synaptic oxytocin receptors stimulate 5-HT release from serotonergic nerve terminals arising from dorsal raphe. Thus, a reduced activity of this pathway might have important implication in the pathophysiology of autistic behavior. As a matter of fact, a recent study shows that 5-HT and oxytocin cooperate in the NAc and their combined activity is crucially involved in social reward, a mechanism disrupted in autism (Dölen et al., 2013).

## 5-HT_7_ receptors as a possible novel therapeutic target in autism spectrum disorders

Little is known about a possible malfunction of 5-HT_7_ receptors in ASD. A single base polymorphism was detected in the gene coding for the 5-HT_7_ receptor, giving rise to two different alleles; however, analysis of transmission disequilibrium in autistic patients did not evidence any correlation of either allele to autism (Lassig et al., 1999). The expression level of 5-HT_7_ receptors in the brain of autistic patients has not been specifically investigated and no information is presently available about the functional properties of 5-HT_7_ receptors in animal models of autism.

Although a dysfunction of 5-HT_7_ receptors has not been causally linked to ASD, we propose that 5-HT_7_ receptor ligands might be considered as valuable pharmacological tools in ASD. This suggestion is mainly based on our findings but is also supported by other studies addressing the role of 5-HT_7_ receptors in behavioral flexibility and repetitive behavior, as discussed below. We have shown that 5-HT_7_ receptor activation reverses mGluR-LTD in Fmr1KO mice (Costa et al., 2012a), a model of Fragile X Syndrome also considered as an animal model of autism (Bernardet and Crusio, 2006; Pietropaolo et al., 2011). This result might open new perspectives for therapy if pharmacological activation of 5-HT_7_ receptors, besides correcting the most prominent synaptic defect in the mouse model of FXS, is also able to rescue the symptoms of FXS such as learning deficits, epilepsy and autistic behavior.

A typical feature of ASD is a reduced behavioral flexibility, i.e., a reduced ability to replace a previously acquired rule with a new one, in adaptation to a new environmental context. Behavioral flexibility depends on brain circuits involving pre-frontal cortex (Floresco et al., 2009; Wolfensteller and Ruge, 2012; Logue and Gould, 2014) and can be evaluated in animals as well as in humans using an attentional set-shifting task (Birrell and Brown, 2000; Colacicco et al., 2002). In this test, rodents are trained to discriminate between different stimuli (odor, surface texture, digging medium) as a cue to obtain a reward. After a training period, the rules are changed and animals must learn that a previously relevant cue has become irrelevant and vice-versa. Using attentional set-shifting protocols, recent studies have shown an involvement of 5-HT_7_ receptors in behavioral flexibility, especially in conditions of stress (Nikiforuk, 2012; Nikiforuk and Popik, 2013). Rats exposed to restraint stress showed reduced behavioral flexibility; administration of amisulpride, an atypical antipsychotic drug and a 5-HT_7_ receptor antagonist, reversed the restraint-induced cognitive inflexibility and also improved attention in unstressed animals. Administration of the 5-HT_7_ receptor agonist AS-19 abolished the pro-cognitive effect of amisulpride. The same authors have shown that behavioral inflexibility induced by another protocol (ketamine administration) was reduced by amisulpride and by the 5-HT_7_ receptor antagonist SB-269970 (Nikiforuk et al., 2013). These results suggest that a lack of behavioral flexibility is associated with increased activation of 5-HT_7_ receptors. In this respect, antagonists rather than agonist of 5-HT_7_ receptors might be beneficial to correct the reduced behavioral flexibility observed in ASD. However, further studies are necessary to test the effects of 5-HT_7_ receptor agonists and antagonist on cognitive flexibility in different pathological conditions. In particular, it would be useful to study the relation between altered synaptic plasticity and behavioral flexibility in different models of intellectual disability and autism. For example, the Fmr1 KO mouse model of Fragile X Syndrome exhibit an abnormally enhanced mGluR-LTD (Huber et al., 2002) and show autistic features and reduced behavioral flexibility (Casten et al., 2011). On the other side, mGluR-LTD is abnormally reduced in other mouse models of syndromic and non syndromic forms of autism, which also show reduced behavioral flexibility. We are currently studying if systemic administration of a 5-HT_7_ receptor agonist can rescue learning deficits and altered behavior in Fmr1 KO mice. Our hypothesis is that 5-HT_7_ receptor activation, although reducing behavioral flexibility in wild-type animals (Nikiforuk, 2012; Nikiforuk and Popik, 2013), might restore behavior and thus be beneficial in Fragile X syndrome, a condition in which mGluR-LTD is exaggerated.

Repetitive and stereotypic behavior is considered a core symptom of ASD. A behavioral study has shown that the stereotypical behavior of marble burying, an experimental protocol used to evaluate anxiety and obsessive-compulsive behavior, was reduced in 5-HT_7_ KO mice and in wild-type mice following pharmacological blockade of 5-HT_7_ receptors (Hedlund and Sutcliffe, 2007). The authors suggest that this result might also be due to the anxiolytic effect of 5-HT_7_ receptor blockade (Hedlund, 2009).

Co-morbid symptoms of ASD, observed only in a fraction of patients, include sleep disorders, anxiety, depression, epilepsy, attention deficit and hyperactivity disorder (Spooren et al., 2012). As pointed out in the previous paragraphs, 5-HT_7_ receptors are crucially involved in the regulation of the sleep/wake cycle, exert either pro- or anti-convulsant effects in different models of epilepsy and play an important role in mood control (reviewed by Bagdy et al., 2007; Matthys et al., 2011). Since 5-HT_7_ receptor inactivation or blockade exert anxiolytic effects (see above), 5-HT_7_ receptor activation is likely to enhance the level of anxiety, thus might be expected to reduce hyperactivity. In line with this hypothesis, in highly active rats showing explorative behavior and low levels of anxiety, the expression level of 5-HT_7_ receptors in the thalamus and in the hippocampus was significantly lower than in low-activity rats (Ballaz et al., 2007b). Accordingly, another study shows that microinjections of the 5-HT_7_ receptor agonist AS-19 into the NAc decreased ambulatory activity in the rat (Clissold et al., 2013).

Other publications instead show that systemic administration of the 5-HT_7_ receptor agonist LP-211 enhanced locomotion in wild-type mice (Adriani et al., 2012) and exerted anxiolytic effects, enhancing the exploratory attitude, in a rat model of hyperactivity and attention deficit (Ruocco et al., 2013). Therefore, it would be interesting to further investigate the role of 5-HT_7_ receptors on different types of anxiety-related behaviors.

### Concluding remarks

Central 5-HT_7_ receptors modulate neuronal excitability and synaptic function and are important physiological modulators of learning and mood. Based on these properties, 5-HT_7_ receptors have been proposed as a pharmacological target in cognitive impairment, depression and epilepsy.

Core and co-morbid symptoms of ASD involve a disruption of several functions, some of which are physiologically regulated by 5-HT_7_ receptors. In light of this, it would be interesting to investigate the role of 5-HT_7_ receptors on different features disrupted in ASD. Cognitive functions are likely to be enhanced by 5-HT_7_ receptor agonists; on the other side, behavioral flexibility, hyperactivity and epilepsy might benefit from blockade of 5-HT_7_ receptors. In many cases, predictions of a possible outcome by pharmacological manipulation of 5-HT_7_ receptors are complicated by heterogeneous data present in literature and a direct investigation would be necessary.

## Conflict of interest statement

The authors declare that the research was conducted in the absence of any commercial or financial relationships that could be construed as a potential conflict of interest.
